# High Temperature and Hygiene in Dress

**Published:** 1904-07-30

**Authors:** 


### High Temperature and Hygiene in Dress.

"Summer," wrote Horace Walpole, "has set in with its accustomed severity," and there can be no doubt, if we may for a moment deviate from the stately language of an English classic to that of a Transatlantic penny-a-liner, that the "heat-wave" of the now current July has been one of unusual suddenness and intensity. We even hear of numerous deaths due, or at least attributed to, the heat; although we may, perhaps, derive some comfort from the consideration that they are mostly said to have occurred in France, or in America, or in some other country sufficiently remote to place difficulties in the way of immediate verification. To the heights attained by certain thermometers there has apparently been no limit; but it is necessary to remember that many of the instruments sold under the name are mere toys, in no way to be relied upon for accurate indications, and that they are often so placed as to be exposed to quite artificial conditions. We are the fortunate possessors of two thermometers by the same maker, or at least stamped with the same name, which hang side by side in the same apartment, and their indications never differ by less than five degrees, and sometimes by more. Making due allowance for such circumstances, and for the incessant demand for startling "pars" which proceeds from the offices of inferior newspapers, we must still allow that the weather has been very warm, and we must once more express our wonder that, among a people commonly supposed to be above all things practical, conditions of annual recurrence are not better provided against than is usually the case amongst ourselves. The books about the elements of science which were prepared for the children of 60 years ago often contained an account of an experiment intended to show the different heat-absorbing powers of different colours. The juvenile readers were told to procure a few strips of black and coloured cloth and to place them upon snow in sunshine, in order to see that the black cloth would rapidly thaw the snow and sink to the level of the surface upon which it rested, that the white cloth would not sink at all, and that various colours would sink to different degrees of depth. Quite lately a French *savant* is said to have coated the bulb of a thermometer with black paint, with the result that it indicated 17 degrees more of heat than one adjacent to it which had not been thus treated; and the experiment has been gravely described in "pars" as one which has added something to the sum of human knowledge. We do not pledge ourselves to any precise number of degrees, whether of Fahrenheit or, as with a French thermometer they presumably would be, of Centigrade; but if the *savant* who tried the experiment was surprised at the result we can only say that we are sorry for him. Whether it be new or old, however, it has none the less its practical applications; and one of them is that hot weather should be the signal for laying aside dark-coloured clothing. We have seen of late years a considerable step in this direction by the growing popularity of the straw hat; but what is really wanted, in the interests of health and comfort, is light-coloured body clothing. The case is one in which we would venture to make an appeal to the clergy; for we believe that the supposed "respectability" of dark or black clothing is solely due to their example in wearing it. We have never been able to understand why this example should be set; inasmuch as the colour is also popularly associated with a character the very reverse of respectable, against whose works the clergy are supposed to strive; while the future conditions which it is their business to assist us in securing are equally associated with white or shining garments. If an archbishop would but set the example of wearing white drill he would deserve the eternal gratitude of the nation; and the directors of banks and great companies would hardly less be public benefactors if in hot weather they would sanction, or even enjoin, the appearance of their clerks in garments which might be perfectly decorous in cut, but made of some light-coloured material easily amenable to washing or cleaning. Black clothes not only absorb a quantity of heat, and convey it, to his great discomfort or injury, to the wearer, but they are, as a rule, saturated with atmospheric and personal dirt, and therefore noxious alike to the owner and to those who come into close juxtaposition with him. A stout reverend gentleman, perspiring copiously in black clothes of some antiquity, is not an agreeable neighbour in an omnibus or a railway carriage, and his presence, unfortunately, is less uncommon than might be desired. There will usually be at least four weeks in every English summer in which dark clothing is totally unsuitable to the climate, and if the clergy will not lead the way to its abandonment we may at least appeal to the medical profession. Why a physician or surgeon should so far yield to custom as to clothe himself in a manner which he would be the first to admit was at once uncleanly, uncomfortable, and unwholesome, is a matter which, as Lord Dundreary would say, "No fellah can be expected to understand."

